# Molecular Assessment of Plasma Concentrations of Selected Adipokines and IL-8 in Horses with Back Pain and Comorbid Asthma—Based on Clinical Cases

**DOI:** 10.3390/ani15030310

**Published:** 2025-01-22

**Authors:** Beata Nowicka, Izabela Polkowska, Paulina Zeliszewska-Duk, Anna Torres, Mariusz Duk

**Affiliations:** 1 Department and Clinic of Animal Surgery, University of Life Sciences in Lublin, Głęboka 30, 20-612 Lublin, Poland; iza-polkowska@tlen.pl; 2 Department of Horse Breeding and Use, Faculty of Animal Sciences and Bioeconomy, University of Life Sciences in Lublin, Akademicka 13, 20-950 Lublin, Poland; paulina.zeliszewska@up.lublin.pl; 3 Department of Pediatrics and Adolescent Gynecology, Medical University of Lublin, Chodzki 4, 20-093 Lublin, Poland; anna.torres@umlub.pl; 4 Department of Electronics and Information Technology, Faculty of Electrical Engineering and Computer Science, Lublin University of Technology, 20-618 Lublin, Poland; m.duk@pollub.pl

**Keywords:** back pain, asthma, adipokines

## Abstract

Similarly, in humans and horses, thoracic and lumbosacral back pain cause more disability and work interruptions worldwide than any other disease. Given that there are few effective treatments for back pain in humans and animals, primary prevention strategies and a reduction in pain factors may be crucial. In the analysed data obtained for the horses studied, the pattern of changes in adipocytokine concentrations, including resistin, visfatin and leptin, was noted for those with back pain compared to the control animals. Concentrations of selected adipocytokines in horses from the back pain group were different in animals with a coexisting diagnosis of asthma and back dysfunction. Very few studies are available on adipokine concentrations in horses. No information was found in relation to back pain and asthma in these animals. In humans, correlations of back pain and asthma with concentrations of selected adipokines have been described.

## Simple Summary

Tissues (for instance, muscle, adipose tissue but also the others) which are metabolically active, can be sources of protein hormones. Many adipocytokines are pro-inflammatory in nature and exacerbate clinical symptoms, including pain, in a specified region. In case of humans, correlations of back pain and asthma with concentrations of selected adipokines have been identified. The fact that adipocytokines play an important role in different types of pain can be concluded from the observations, however, the underlying mechanisms is unclear. Therefore, it seems crucial to define their action in specific disease entities. Back pain in horses is a disorder with a multifactorial aetiology, so seeking for and attempts to identify new factors involved in their pathogenesis may contribute to the introduction of preventive measures and changes in treatment.

## 1. Introduction

There is increasing evidence for the association of musculoskeletal pain with systemic metabolic processes in weight-bearing as well as non-weight-bearing areas [1]. Metabolically active tissues (muscle, adipose tissue, synovial membrane, blood, smooth muscles of the vascular wall and others) can be sources of protein hormones. There is also the endo, exo, and paracrine distribution of peptides. Many cytokines and adipokines that are pro-inflammatory in nature exacerbate clinical symptoms, including pain, in a given region [2]. Altered excitation thresholds and peripheral nerve responses to stimuli may be a consequence of inflammation, leading to increased peripheral and central nervous system responses [3,4]. Due to their action, they are said to facilitate and inhibit inflammation at the same time.

Regardless of the species, the dynamic stability of the musculoskeletal structures of the spinal region is required in any sporting discipline. This allows for free movements of the limbs and axial skeleton as well as a response to destabilising forces. This optimises the energy expended and reduces the risk of injury [5,6,7,8,9].

The main postural muscle in humans and horses is the Musculus multifidus, the main muscle responsible for providing intersegmental stabilisation of the spine [7,8]. Preliminary biomechanical modelling may suggest a comparable function of the multifidus in horses to its function in humans [6,9]. Anatomical, biomechanical, and histological relations in the thoracic and sacro-lumbar (TH, L-S) segments, including the fascia, also appear similar [8,9,10,11].

As in the case of human patients with TH and L-S pain, there are often no absolute definitive radiographic discoveries exclusive to making the diagnosis of back pain. Back pain shall be characterised as a generalised finding, and back disease in horses may result from multifactorial aetiology that potentially involve the musculoskeletal system and fascia [10,11]. Clinical signs may arise from various factors: biological, environmental, and/or mechanical. Back diseases in horses have a multifactorial aetiology and involve diseases of the musculoskeletal system, including the fascia. Horses’ back diseases are mainly connected with osseous ligament- and joint-related injuries or chronic diseases, soft-tissue lesions, and lameness- and tack-associated problems [12,13,14,15,16]. The diagnosis of back disease and back pain can be difficult and is most often preceded by the elimination of other diseases of the musculoskeletal system, including the limbs [17,18,19]. Back disorders can be primary or secondary, and are manifested by pain (back pain, BP) [20,21].

**Figure 1:**
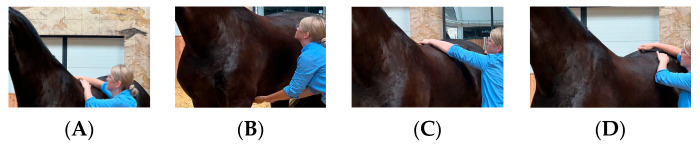
A clinical examination of the horse’s back. Location of pain, dysfunction, and the assessment of mobility of individual segments, respectively: (**A**,**B**) thoracic; (**C**,**D**) sacro-lumbar region.

Clinical examination allows for a palpable diagnosis of pain to be made, taking into account the lack of spasmodic response, excessive response to pressure, and reduced range of mobility, including reduced flexibility of the thoracolumbar spine (Figure 1). Consideration is given to the animal’s responses, including during the clinical examination, and observed behavioural abnormalities, such as when under saddle. Abnormal dorsal musculature, soreness, and poor hind limb impulsion observed during movement may be noted during the examination [17,22].

In animals and humans, asthma is described as a chronic inflammatory airway disease. The resulting cellular components lead to reversible airway obstruction, causing respiratory symptoms, such as wheezing, coughing, and dyspnoea [23,24].

An increased mucous production and bronchospasm with thickening of the bronchial walls are the results of equine asthma, which occurs due to triggers that include exposure to conditions with high levels of dust and mould [24,25,26]. Diagnosis is based on patient history and the clinical manifestation of disease, which most commonly includes a chronic reduction in athletic performance lasting at least 3 weeks in terms of duration. Supportive diagnostic findings in cases of equine asthma involve a lower airway endoscopic evaluation combined with bronchoalvelolar lavage that reveal increased mucous production with a neutrophilic inflammatory exudate. Similarly to humans, asthma in horses is a heterogeneous disease with two currently recognised phenotypes: mild/moderate EAS and sEAS. Mild/moderate EAS has been recently reviewed [8]. There are several forms of asthma: severe and mild/moderate equine [27]. It is reported that mild asthma affects as many as 80% of some athletic equine populations [28]. 

In recent years, a number of studies have revealed the important role of adipocytokines (including selected resistin, visfatin, leptin, and IL-8) in diverse physiological processes, inflammation and immune functions, glucose and lipid metabolism, and musculoskeletal diseases [29,30,31].

Resistin is a cysteine-rich peptide in both humans and animals, produced by adipocytes, macrophages, monocytes, leukocytes, and mature osteoblasts [32,33,34]. In skeletal muscles, resistin interferes with insulin-stimulated glucose uptake and affects the glucose levels produced in the liver [35,36,37]. By antagonising the action of insulin, it reduces glucose uptake in adipocytes, muscle cells, and other tissues [38,39]. Plasma resistin levels were shown to be unchanged in horses with moderate inflammation of the small intestine, while its levels were elevated in the severe form of the disease. The correlation of this adipokine was studied in horses between obesity and insulin secretion and inflammation, as well as in metabolic disorders [40].

Visfatin and other biomarkers characterising inflammation were found to be associated with bone turnover and the ability to predict radiographic progression in patients with ankylosing spondylitis [40]. Its secretion was localised in many tissue types, including the musculoskeletal system [41].

Leptin is a protein hormone with multidirectional effects, with receptors identified in degenerated regions of rat and human nucleus pulposus [42]. The stimulation of mouse myotubules with leptin was identified to increase glucose uptake, whilst the overexpression of leptin in a mouse model increased insulin sensitivity [43]. A rat skeletal muscle cell line’s stimulation with recombinant leptin decreased the phosphorylation of insulin receptor substrate-1 (IRS-1) and impaired glucose uptake, indicating that leptin promotes insulin resistance [42]. Leptin-dependent nitric oxide (NO) production has been described in relation to the pathophysiology of human’s lumbosacral pain [40,44]. The interaction between leptin and selected interleukins, alone and in combination with inflammatory mediators, increases the rate of NO production.

Additionally to adipose tissue, skeletal muscle has been identified as an endocrine organ, with its cells producing cytokines with potential hormonal effects. Cultured skeletal muscle cells can secrete interleukin-8 or tumour necrosis factor (TNF)-α, among others [45].

It remains unclear what the exact significance of adipokines in specific disease entities is, as well as the consequences of their mutual interactions in horses [35]. Recent studies show that, in humans, adipocytokines can shape the body’s response in the pathogenesis of musculoskeletal diseases of diverse aetiologies, with variable values at different stages, with a consequential effect on a diverse response to treatment [45,46,47,48].

To the authors’ knowledge, there are no data for horses regarding the concentration of selected adipokines and back diseases, and for animals diagnosed with asthma. Therefore, research in human medicine served as a reference point for the topic under consideration.

The literature available does not contain any studies on the concentration of selected adipokines in horses diagnosed with back pain or asthma. It is important to take into account co-occurring diseases when qualifying animals to study groups, because the results of adipokine concentration in horses examined in terms of diseases of the musculoskeletal system may be influenced by diseases in the respiratory system. The possible impact of the diseases in question on the results obtained in relation to the concentration of resistin, visfatin, leptin, and IL-8 is considered.

## 2. Materials and Methods

### 2.1. Group Eligibility Study Design

The group eligibility study included a detailed veterinary medical history, nutrition, and guidance on the maintenance and use of the animal.

A clinical examination was performed, including a BCS assessment of the horses’ condition (muscling, fat content) based on the BCS (Body Condition Scoring) in order to determine the examined horse’s body fat content. The neck, back, ribs, and pelvis areas were assessed on a scale from 0 to 5 on a range of fat to muscle mass. The assessment was based on a scientific study by Henneke D.R et al. [49]. To obtain an overall measurement, the results were averaged. For horses with excessive body fat, the data obtained were not utilised for further analysis. The tests and examined parameters included: posture, conformation, assessment of joint angulation, hoof cup conformation, muscling, maintenance of the animal, palpation examination (head, neck, back, thoracic bones, and pelvic bones, including accessible tendon structures), hoof checkup to exclude or confirm pressure reactions in the pastern and hoof sole, flexion tests and the verification of the range of mobility in the different spinal segments and the atlanto-occipital joint, passive pro- and retraction tests, and flexion tests for the thoracic and pelvic limbs. Imaging tests were also performed in justified cases, as were laboratory tests.

To reduce the number of variables affecting the outcome of the clinical analysis and to maintain similar conditions, a single person with a level of training and experience relevant to making a correct diagnosis (DVM, PhD veterinarian) performed all stages of the examination of all horses.

The evaluation of back dysfunction in each horse was observed on the basis of established procedures, which was based on the analysis obtained during the clinical examination of the horse. This included palpation, passive flexion tests (lateral, dorsal, and ventral), and the mobility of the cervical, thoracic, sacro-lumbar, and pelvic regions. It also included a visual and palpation assessment of the mentioned spinal regions, including the reaction to touch, and pressure in the tested areas. Animals were also tested in motion: on soft and hard surfaces, in a straight line and in a circle, whilst walking and trotting and, when in doubt, under saddle.

In horses with back pain, based on the history taken, asthma was noted as a comorbid condition, which was identified as a recurrent factor in a sufficient number of patients that were then analysed (based on a detailed interview with the owner and an analysis of the medical records).

The analysis was based on a retrospective study, in which the horses were classified into a group with back pain, a group with back pain with asthma, and a group of healthy horses (no asthma or back pain). All horses classified in the BP + A group had a history of equine asthma diagnosed by BAL and clinical examination.

The diagnosis of this disease in humans is primarily based on a detailed history and clinical examination. It is useful in horses, but the gold standard is still to perform a bronchoalveolar lavage fluid (BALF) examination [27].

### 2.2. Animal Eligibility Criteria for Each Group

The retrospective study included 28 horses, all patients of the University of Life Sciences in Lublin, the Department of Animal Surgery. In order to limit the influence of additional factors on the results, the following exclusion criteria were applied for each group: horses qualified for the BCS 0–1 and 4–5 ranges (Body Condition Scoring of horses), the presence of other chronic diseases, mating time, and an age below 4 years and above 20 years.

A total of 11 animals were included in the study group (BP), based on the following criteria: diagnosis of back pain, an age between 4 and 20 years, a BCS range of 2–3, no treatment in the 2 months preceding the study, and no other general diseases of a chronic nature.

After analysing the available data, a further group of horses was selected with the following diagnoses: back pain and coexisting asthma (BP + A). Asthma was confirmed on the basis of clinical and veterinary history (results kept by the animal owner and treatment history). A total of 9 horses were included in the group.

The control group (C) consisted of 9 horses without any signs or diagnosis of BP and without any signs or diagnosis of asthma. Animals in the control group were qualified based on the following criteria: (i) absence of BP diagnosis and lameness after examination performed for routine control, (ii) prophylaxis or check-up, (iii) according to the will and knowledge of the owner. The inclusion criteria were: (i) age between 4 and 20 years, (ii) BCS range 2–3, (iii) no diagnosis of BP and/or asthma, (iv) no signs of comorbidities, and (v) no treatment in the 4 months preceding the study.

### 2.3. Method Used to Assess Adipokine and Interleukin 8 Levels

The study material consisted of 10 mL of peripheral blood collected from the external jugular vein into tubes containing EDTA during routine examinations. Then, the blood samples were subjected to processing in the laboratory, i.e., cooling to 4 °C and centrifugation for 10 min at 1500 rpm. The resulting plasma was collected, portioned, and stored at −80 °C until further indication.

Levels of leptin, resistin, visfatin, and interleukin 8 were evaluated using a magnetic sphere-based method with commercially available Multiplex kits and a Luminex^®^ MagPiX automated analyser (Luminex, Austin, TX, USA).

This technology allows for the determination of concentrations of multiple different molecules in a single test sample. In the study presented here, leptin, resistin, visfatin, and interleukin 8 were determined simultaneously in a single plasma sample. Luminex technology is based on the use of polystyrene or magnetic microspheres. In this study, measurements were obtained by identifying individual magnetic spheres containing a fluorescent dye. The antibodies on the surface of the spheres have an affinity for the individual antigens, enabling the determination of the markers under study.

### 2.4. Experimental Procedure for the Assessment of Adipokine Concentrations

The plasma samples were diluted in a suitable manner and added to microspheres coated with capture antibodies, which sequentially bonded with the relevant substances, enabling their determination. Subsequently, the mixture was supplemented with biotinylated detection antibodies binding to the specific markers under determination, which formed an antigen–antibody complex. In the final step, the reaction mixture was incubated with the streptavidin–phycoerythrin complex to complete the reaction on the surface of each sphere.

**Figure 2:**
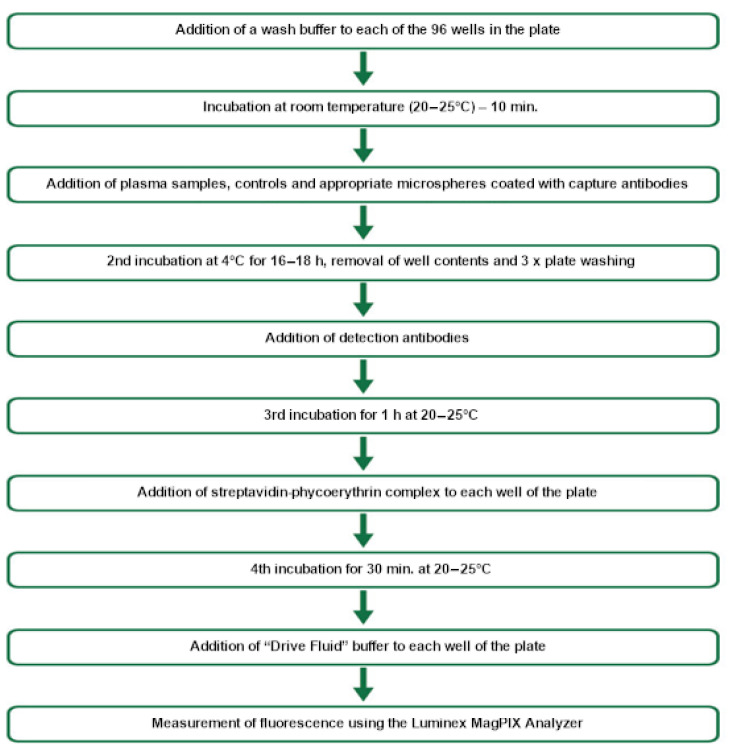
A diagram of the determination of adipokine and interleukin 8 concentrations.

Quantitative assessments were made based on the intensity of the fluorescent signal to obtain the final result. Concentrations of selected adipokines and interleukin 8 were assessed according to the manufacturer’s protocol. Concentrations were calculated according to a five-parameter logarithmic curve using xPonent 4.2. A magnetic sphere-based method was used to assess the concentrations of selected adipokines (leptin, resistin, visfatin, and interleukin 8). The assessment was performed using commercial Multiplex kits and MagPiX, an automated analyser from Luminex^®^. The technology used allowed for concentrations of different molecules to be determined in a single test sample, including resistin, visfatin, leptin, and interleukin 8 (Figure 2).

### 2.5. Statistical Analysis

The results of the analysis of resistin, visfatin, leptin, and interleukin 8 concentrations are summarised in tables. The data were organised taking into account the division of the tested horses into three groups: horses with back pain, horses with back pain and respiratory problems, and horses from the control group. The data prepared in this way were entered into the Statistica 13.3 program, which was used to determine the basic descriptive statistics characterising the concentrations of individual factors in the blood of the tested horses. Descriptive statistics included parameters such as: mean, median, minimum, and maximum values, as well as the standard deviation of individual factors in the study groups. Based on the obtained analyses, box charts were prepared to illustrate the obtained results. Statistical analyses were performed using GraphPad Prism 9 (GraphPad Software, Boston, MA, USA), as described in each respective figure legend.

## 3. Results and Discussion

### 3.1. Results

There were four times more geldings than mares in the group of horses with a diagnosis of back pain, while stallions accounted for only 7.69% of the group. In horses with comorbid asthma, there were twice as many mares as geldings, while in the control group, mares, geldings, and stallions made up equal proportions of the group. The average age in all groups was similar and in the range of 10–11 years. All groups consisted mainly of the Polish noble half-breed horses (90%). Considering the Body Condition Scoring (BCS) indices, there were more than three times as many horses in the group of horses with a diagnosis of back pain (BP) for BCS 2 than in the BCS 3 group. There were similar proportions of BCS 2 and BCS 3 animals in the group of horses with back pain and asthma (BP + A) and the control group (C) (Table 1). Based on the data collected from the horses owners and documentation shared by them, eight horses (90%) from group BP + A had a mild form of asthma, and only one of them had a severe type of asthma (10%).

When analysing the available literature, it was found that there was no clear effect of obesity on plasma resistin levels in both humans and animals, including horses. In our retrospective study, data from overly fat or overly lean horses were not qualified for further analysis (BCS 0, BCS 1, BCS4, BCS5).

**Figure 3:**
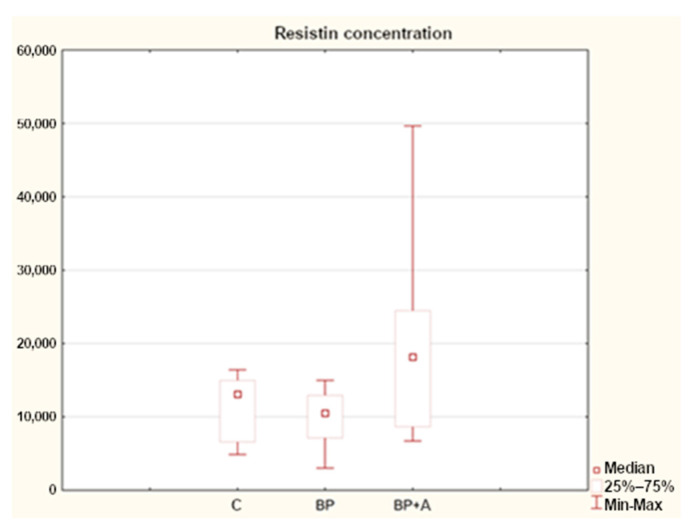
Resistin concentration in horses in the study groups (ng/mL). C—control group; BP—with back pain; and BP + A—with back pain and asthma.

Statistical analysis of the data showed differences in resistin concentrations among the horses in the study groups. In those with a diagnosis of back pain, the median resistin concentration was lower compared to those in the control group. Despite the lack of differences in fatness, we noted the highest levels of resistin in animals with asthma and back pain, relative to the above-mentioned groups (BP +A, BP) (Figure 3).

The median resistin concentration in the control group of the horses (C) was 13,078.77 ng/mL, whereas in the group with back pain (BP) it was lower, at 10,528.653 ng/mL. In the group of horses with back pain and asthma (BP + A) it was 18,169.29 ng/mL, which was higher than in the group with a diagnosis of back pain only (Figure 3).

**Figure 4:**
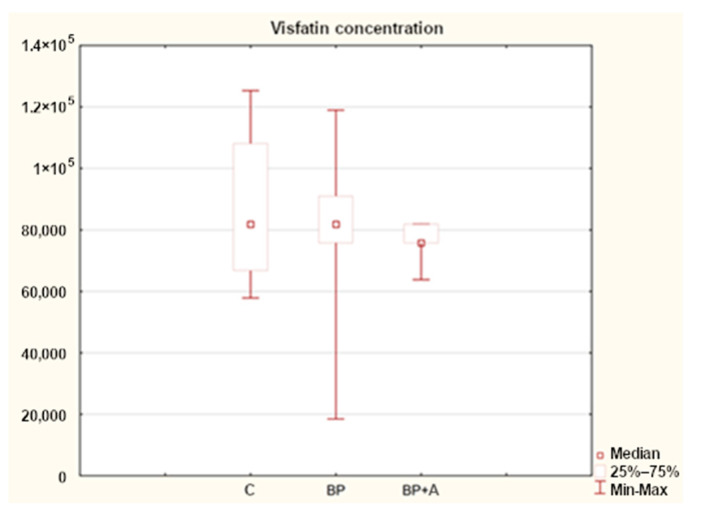
Visfatin concentration in horses in the study groups (ng/mL). C—control group; BP—with back pain; and BP + A—with back pain and asthma.

Statistical analysis showed differences in the concentration of visfatin in horses in all the study groups. The median concentration of visfatin in healthy horses (C) was 81,838.95 ng/mL; in the group with back pain, it was 81,817.42 ng/mL; and in the group with asthma and back pain, it was 75,758.13 ng/mL (Figure 4).

**Figure 5:**
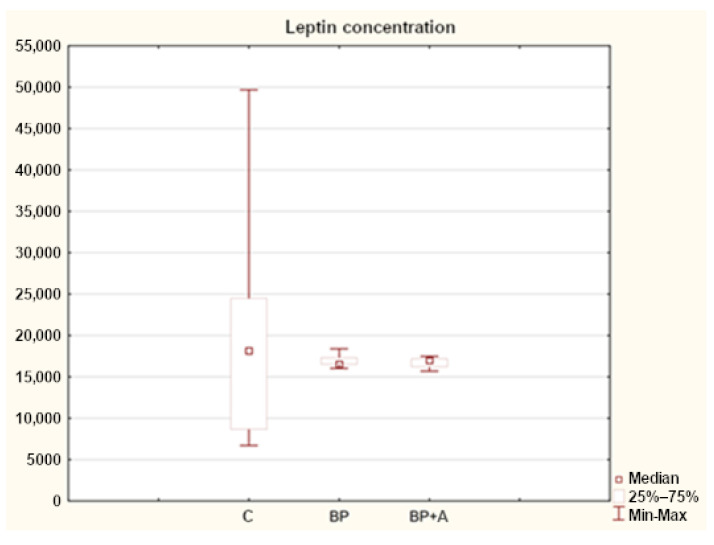
Leptin concentration in horses in the study groups (ng/mL). C—control group; BP—with back pain; BP + A—with back pain and asthma.

Statistical analysis showed differences in the leptin concentrations for the horses in the study groups. The median leptin concentration in the healthy horses (C) was 18,169.29 units, compared to 16,522.57 ng/mL (BP) in the group with back pain and 17,003.63 ng/mL (BP + A) in the group with respiratory problems (Figure 5).

**Figure 6:**
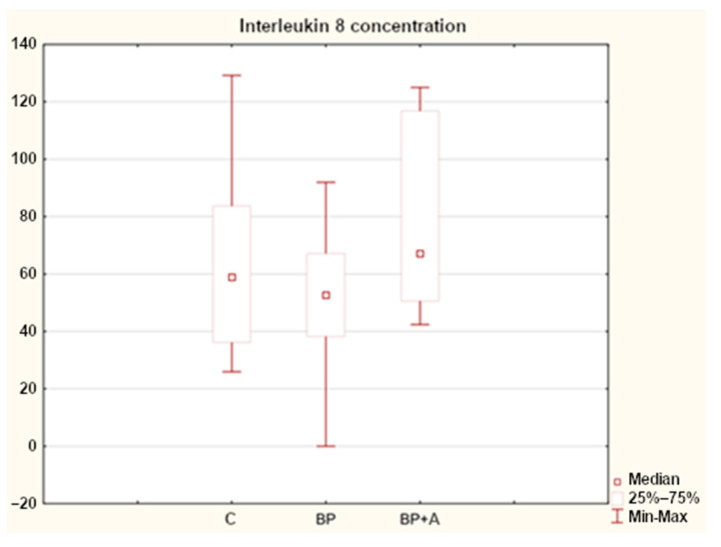
Interleukin 8 concentration in horses in the study groups (ng/mL). C—control group; BP—with back pain; BP + A—with back pain and asthma.

For interleukin 8 concentrations, a difference was found in all the study groups. The control group median was 58.897 ng/mL (C); it was 67.143 ng/mL (BP + A) in the group with back pain and asthma, and it was lower for the group with back pain, at 52.697 ng/mL (BP) (Figure 6).

### 3.2. Discussion

The mechanisms of action for adipocytokines in the presence of pain are complex. Adipocytokines have been shown to be associated with multiple signalling pathways, such as serotonin and inflammatory cytokines; however, the underlying mechanisms of action remain unclear. Potentially, it can be assumed that adipocytokines play an important role in the treatment of pain and will have applications in its treatment in the future.

Resistin, depending on its concentration and conformation, was observed to have reversible effects, with simultaneous consequences on the physiological and pathological processes in relation to diverse tissues [50,51,52]. This adipokine has been tentatively proposed as a novel biomarker of pain intensity, but its mechanism of action remains unclear. Hozumi et al. showed that resistin levels correlate with the severity of postoperative pain, which has been linked to inflammation in postoperative wounds [53]. Resistin plays an important role in the treatment of pain associated with osteoarthritis; however, whether this is related to function or pain is still a matter of debate. A role for resistin in humans has been described in stress biology and, in some cases, as a diagnostic biomarker in assessing disease as well as treatment outcomes in joint disease [51,52]. It has also been noticed that resistin interferes with glucose uptake, which is insulin-stimulated in skeletal muscles [37]. It also affects the regulation of glucose levels by stimulating glucose production in the liver [38,39]. A slight increase in serum resistin levels was reported in patients with rheumatoid arthritis (RA), while its levels were significantly higher in the synovial fluid [54].

In horses, it was shown that plasma resistin concentrations were unchanged in those with moderate ID, but its levels were elevated in the severe forms of the disease [54].

Studies on resistin by Jamaluddin and colleagues show its involvement in important mechanisms of asthma pathogenesis, including the induction of inflammation, angiogenesis, and smooth muscle cell proliferation [55]. In the work by Ballantyne et al., the plasma resistin level was a predictor of asthma risk. This study revealed that resistin levels were higher in those with more severe disease relative to those with mild and moderate disease and compared to the control group [56,57].

In our study, the median resistin concentrations were lower in horses with a diagnosis of back pain compared to those in the control group. The highest levels of resistin were recorded in the group of animals that had a diagnosis of asthma with comorbid back pain (Figure 3).

In humans, Franco-Trepat et al. considered visfatin in the context of musculoskeletal pathologies (MSP), including osteoarthritis (OA) and osteoporosis (OP). Both disease entities are associated with a number of risk factors, such as ageing, metabolic changes and inflammation [58]. Revelo and Ye et al. confirmed using in vivo models that inflammatory environments were associated with higher levels of circulating visfatin [59,60]. Rechardt et al., in their study, demonstrated an association between visfatin and the severity of upper limb pain in humans [61]. In their research paper, Guzik et al. demonstrated the stimulatory effect of inflammation on resistin and visfatin secretion [62]. Currently, visfatin is presented as a promising marker with applications in pain management therapies. At the cellular level, blocking the calcium channel using pregablin utilised the release of visfatin, inducing anti-inflammatory and antioxidant effects [63].

Visfatin induces osteoarthritis pain by stimulating the chondrocytes to release neuronal growth factor (NGF) [64]. In contrast, the role of visfatin in other animal models of pain has not been clarified, and further clinical studies are needed to confirm the role and therapeutic potential of visfatin in the occurrence and treatment of other disease entities.

In a study by Machura et al., a correlation was observed between atopic asthma and low serum visfatin levels in children [65]. Magron et al. found increased serum visfatin levels in obese children diagnosed with asthma [66]. The literature data support the pro-inflammatory nature of visfatin in relation to the induction of pro-inflammatory cytokine responses in the development of asthma [67].

In our analyses, we observed differences in the concentrations of visfatin between horses in the control group (C), where it was the highest, and the group with back pain and asthma as a comorbidity. In this group, the visfatin value was the lowest (BP + A) (Figure 4).

### 3.3. Leptin

Leptin is widely regarded as a pro-inflammatory adipokine. Xibillé-Friedmann et al. showed that, in patients starting RA treatment, the serum leptin levels were associated with the patient’s clinical status [68]. Chimenti et al. suggested the potential for the therapeutic use of leptin through its effect on insulin levels, which could have a beneficial effect on the inflammatory parameters and cardiovascular function in RA patients [69]. Lim et al. showed that leptin causes neuropathic pain in chronic sciatic nerve injury [59,60]. In a model of preganglionic cervical root avulsion (PCRA) [69,70] and neuropathic pain, a leptin deficiency or blockade attenuated mechanical allodynia. In one experiment, it was possible to reverse this after the administration of exogenous leptin. In contrast, a deficiency in leptin signalling through the central mechanisms significantly attenuated pain behaviour [70,71]. Mirror-image pain is defined as mechanical hypersensitivity on the uninjured side that spreads from the injured nerve [71,72]. Mirror-image pain occurred in myeloid cells in mice lacking a cannabinoid receptor (CB2) associated with leptin signalling [73].

A study of sacroiliac pain in humans suggested an association between the patients’ clinical status and the unitary effects of leptin in combination with inflammatory mediators and concomitant effects on the rate of nitric oxide (NO) production [74].

Studies reported high leptin levels in asthmatic patients. Some studies showed an inverse correlation between leptin and lung function [74,75], as well as weight loss [76,77,78,79,80]. Studies by Leão and Baltirei and their team showed an inverse correlation between leptin and lung function [80]. In humans, studies by Szczepankiewicz et al., Ciprandi et al., and Bantulla et al. found high leptin levels in asthmatic patients [75,76,77,78,79].

In our study analyses, we showed differences in leptin concentrations in horses from the study groups (BP and BP + A) and the control group (C). In the control group, leptin concentrations were higher compared to the group of horses with a diagnosis of back pain, while in the group with back pain and comorbid asthma, the median leptin concentration was higher for both groups, reaching 17003.63 ng/mL (BP + A) (Figure 5).

### 3.4. Interleukin-8

Kaufman and Carl, based on their analysis of the available literature, recognised the possibility of creating targeted and optimal protocols in the treatment of human spinal pain using cytokines, including IL-8. It was noted that IL-8 levels are elevated in the cerebrospinal fluid of patients with chronic lumbosacral pain [80,81,82]. In degenerative diseases within intervertebral disc tissue, IL-8 levels are also elevated compared to non-degenerated tissue [83]. In mouse models, the blockade of the IL-8 receptor, signalling the intervertebral disc degenerative disease and pain, reduced the biochemical tissue features in the area of disc degeneration and the behavioural symptoms of back pain [84,85,86].

The results from Ford et al. showed that asthma patients had elevated levels of inflammatory biomarkers, including interleukin-8 [87]. In people with asthma, differences in IL-8 levels in different asthma phenotypes have been confirmed. In a paper by Gibson et al., it was shown that IL-8 levels are highest in patients with non-eosinophilic asthma compared to patients with eosinophilic asthma and the control group. This makes it possible to distinguish between two patterns of inflammation occurring in people with asthma [88,89]. IL-8 concentrations and chemotactic activity in BALF were greater in horses with asthma, compared with healthy horses, and greater in horses with asthma exposed to hay dust, compared with nonexposed affected horses. An increase in IL-8 concentration accompanied by an increase in the percentage of neutrophils in BALF and the development of clinical signs of asthma were induced in asymptomatic horses with asthma by changing the feed from silage to hay. It has been confirmed that, in horses, IL-8 is necessary in the recruitment of neutrophils, which are involved in the body’s response to asthma [90]. Anisworth and others, in their research, similar to Larso and others, noted an increased production of IL-8 by the monocytes, linking IgE in horses with asthma [88,89,90].

In our analysis of the results obtained for IL-8 concentration, by looking at the median values, one can see lower values for the group of horses with back pain (BP) compared to the control group (C). In contrast, in horses with back pain and asthma (BP + A), the median values are higher compared to the BP group and higher than in the control group.

As observed, the diagnosed musculoskeletal disease entities may have their own adipocytokine profile.

Diseases of the back in horses are disorders characterised with a multifactorial aetiology, so looking for and making attempts to identify new factors incriminated in their disease pathogenesis, including systemic factors, may play a part in prophylaxis or more effective diagnosis at an early stage, and therefore in optimal treatment times and procedures.

The studies carried out were of a preliminary nature; therefore, it would be necessary to expand the profile of research and longitudinally observe the action of adipocytokines, including in diseased animals with asthma co-occurrence.

The available evidence suggests that it is advisable to study the strategies of adipocytokine concentrations in specific disease entities, as this may enable the targeting of their use for treatment related to chronic pain.

The initial and retrospective nature of the research revealed a need to expand research on a larger group of horses with back pain and different forms of asthma, in order to be able to evaluate the influence of certain types of asthma on back pain.

## 4. Conclusions

From the observations made, it can be concluded that adipocytokines play an important role in different types of pain, while the underlying mechanisms remain unclear. Therefore, it seems important to define their action in specific disease entities.

Back pain in horses is a disorder with a multifactorial aetiology, so the search for and attempts to identify new factors involved in their pathogenesis may contribute to the introduction of preventive measures and changes in treatment.

When analysing the median values for all adipokines and the IL-8 studied, with the exception of visfatin, lower values can be observed for the group of horses with back pain compared to horses in the control group (C). In horses with dorsal dysfunction and asthma (BP + A), the median values were higher compared to the group of horses with a diagnosis of back pain only (B).

When qualifying and selecting patients for the study groups in terms of musculoskeletal diseases and the concentration dependence of selected adipocytokines, it seems important to consider the comorbidities related to respiratory diseases.

### Limitations of the Study

The studies carried out were preliminary and retrospective, and, therefore, it is necessary to expand the research profile and to longitudinally observe the action of adipocytokines in diseased animals in specific disease entities and pain pathophysiology, especially with the co-occurrence of asthma. It therefore seems appropriate and valuable to investigate the action of adipocytokines in different models, taking into account specific disease entities.

Logistic and financial constraints limited our ability to achieve the following:

1. An extended radiological diagnostics using ultrasound and/or x-ray imaging of the back;
2. Supplementing with additional markers, including adiponectin, tumour necrosis factor, interleukin-6, and vascular endothelial growth factor.

## Figures and Tables

**Table 1:** The characterisation of the animals in the study group.

Feature	n = 28 (100%)	BP n = 11 (%)	BP + A n = 8 (%)	Control n = 9 (%)
Gender				
mare	32.14	15.38	66.66	33.3
gelding	53.57	7.69	33.34	33.3
stallion	14.29	7.69	0	33.3
Type of asthma	severe (n) 1 mild (n) 7		10% 90%	
Age (years) average		11	12	10
Breed—half-breed horses		90	95	100
other		10	5	-
BCS 2		79	67	53
BCS 3		21	33	47

BCS—Body Condition Scoring—an objective system for assessing physical condition (amount of stored fat and muscle development), using a numerical assessment on a scale from 1 to 5.

## Data Availability

The original contributions presented in the study are included in the article, further inquiries can be directed to the corresponding author.
